# Effects of Ginger Phenylpropanoids and Quercetin on Nrf2-ARE Pathway in Human BJ Fibroblasts and HaCaT Keratinocytes

**DOI:** 10.1155/2016/2173275

**Published:** 2016-01-28

**Authors:** Ermin Schadich, Jan Hlaváč, Tereza Volná, Lakshman Varanasi, Marián Hajdúch, Petr Džubák

**Affiliations:** ^1^Institute of Molecular and Translational Medicine, Faculty of Medicine, Palacký University, Olomouc, Czech Republic; ^2^Department of Organic Chemistry, Faculty of Science, Palacký University, Olomouc, Czech Republic

## Abstract

Quercetin and phenylpropanoids are well known chemoprotective compounds identified in many plants. This study was aimed at determining their effects on activation of Nuclear factor erythroid 2-related factor 2 (Nrf2) antioxidant response element (Nrf2-ARE) signalling pathway and expression of its important downstream effector phase II detoxification enzyme glutathione-S-transferase P1 (GSTP1) in BJ foreskin fibroblasts and skin HaCaT keratinocytes. Cell lines and their corresponding Nrf2-ARE luciferase reporter cells were treated by ginger phenylpropanoids and quercetin for 10 h and the level of Nrf2 activity was subsequently determined. Both, ginger phenylpropanoids and quercetin, significantly increased the level of Nrf2 activity. Subsequent western blot analyses of proteins showed the increased expression level of glutathione-S-transferase P1 (GSTP1) in BJ cells but not in HaCaT cells. Such phenomenon of unresponsive downstream target expression in HaCaT cells was consistent with previous studies showing a constitutive expression of their GSTP1. Thus, while both ginger phenylpropanoids and quercetin have the property of increasing the level of Nrf2 both in HaCaT and in BJ cells, their effects on its downstream signalling were mediated only in BJ cells.

## 1. Introduction

As human skin is repeatedly exposed to excessive level of UV radiation and environmental pollutants; protection of its cells against toxic agents depends on an elaborated antioxidant defense system of enzymes and antioxidants for neutralization of induced reactive oxygen species (ROS), biotransformation and elimination of electrophilic species, and maintenance of redox homeostasis [1, 2]. The involved enzymes are constitutively and inducibly expressed, and, among them, the most significant phase II detoxification enzymes are NADPH oxidoreductase, aldoketo reductase, glutamate-cysteine ligase, and glutathione-S-transferase P1 (GSTP1) [3, 4]. Their inducible expression in skin cells during antioxidant responses is directly or indirectly regulated by the protein transcription factor named Nuclear factor erythroid 2-related factor 2 (Nrf2) [5, 6]. The Nrf2, upon its activation by ROS and electrophilic species, translocates into nucleus, heterodimerizes with the small Maf transcription factor, and activates their expression by binding to the* cis*-acting antioxidant response element (*ARE*) DNA sequence in the promoters of genes encoding them [3, 7].

Under quiescent conditions, the Nrf2 is associated with its cytosolic repressor kelch-like ECH-associated protein 1 (KEAP1) in the Nrf2-KEAP1 complex that promotes its ubiquitination and proteasome degradation [7, 8]. The KEAP1 acts as a redox sensor and different alterations in its structure induced by ROS and electrophilic compounds including oxidative modifications of its cysteine residues (Cys151, Cys273, and Cys288) lead to its dissociation from this complex and activation of Nrf2 [7–9].

Plant phenylpropanoids identified in ginger plant (*Zingiber officinale*), 6-gingerol, and 6-shogaol and their derivatives and plant flavonoid quercetin as well as other dietary plant phenolic compounds are considered as chemopreventive candidates against oxidative stress and cancer due to its property of activating Nrf2-ARE signalling pathway in different types of human cells [9–12]. Their effects on expression of GSTP1 enzyme could be specifically beneficial in biotransformation of ROS and electrophilic species. However, their ability to activate the Nrf2-ARE pathway and expression of GSTP1 enzyme has not been tested* in vitro* in human skin cells, including the immortalized keratinocytes (HaCaT) cells and foreskin fibroblasts (BJ) that are frequently used in various* in vitro* studies. These cell lines are frequently used as a paradigm of skin cells due to their phenotypic stability and well preserved differentiation capacity [13]. The aim of this study is to determine the effects of ginger phenylpropanoids and quercetin on activation of the Nrf2-ARE signalling pathway and expression of phase II detoxification enzyme glutathione-S-transferase P1 (GSTP1) in HaCaT and BJ cells.

## 2. Material and Methods

### 2.1. Cell Lines and Luciferase Cell Reporters

HaCaT cells and BJ foreskin fibroblast were obtained from American Type Culture Collection (ATCC). The stable reporter cell lines were obtained by transduction of HaCaT and BJ cells with Cignal lentiviral particles with ARE reporters using Qiagen kits (CLS-2020L and CLS-013L). Clones were selected and validated according to instructions provided by manufacturer.

### 2.2. Preparation of Ginger Extract

The methanol extract of ginger phenylpropanoids was prepared from commercial powder of rhizome of ginger plant (*Zingiber officinale*) (Vitana). The extraction was done by dissolving 40 g of powder in 200 mL of methanol or distilled deionized water and incubated at room temperature for 24 h with shaking at 120 rpm. After extraction, the solids were removed by centrifugation at 3000 g at 25°C for 10 min and filtration with 0.22 *μ*m membrane filter. Subsequently, the methanol extract was dried out using SpeedVac (Thermo Scientific) at 45°C, dissolved in dimethyl sulfoxide (DMSO), sterilized using 0.22 *μ*m membrane filter, and stored at −80°C.

### 2.3. Ultrahigh Performance Liquid Chromatography Analyses of Phenylpropanoids

The ultrahigh performance liquid chromatography (UHPLC) analyses of ginger extract were performed using Waters UHPLC system as described in previous studies [14–16]. Ginger extract had a concentration of 0.2 mg/mL in 100% DMSO. The separation was carried out on X-Select C18 reversed phase column (3.0 × 50 mm; 2.5 *μ*m) at 30°C using a flow rate of 600 *μ*L/min. Mobile phase A was 0.01 M ammonium acetate in water and mobile phase B was 100% acetonitrile. The elution cycle had a linear gradient from 80% A and 20% B to 20% A and 80% B over the period of 2.5 min. 80% B was maintained over the 1.5 min interval. The column was reequilibrated at 10% B for 1 min. Absorbance of HPLC peaks was measured using a photodiode array (PDA) detector, and the area under each peak (AUC) was used to determine relative compound content and purity. Detection of molecular ions was performed using single quadrupole mass spectrometer (Waters) fitted with an electrospray ionization source for negative and positive ion modes. The atmospheric pressure chemical ionization operated at a discharge current of 5 *μ*A, vaporizer temperature of 350°C, and capillary temperature of 200°C. System is controlled by MASSLYNX (version 4.1) software.

### 2.4. MTT Cytotoxic Assay

The BJ and HaCaT cells were maintained in Nunc/Corning 80 cm^2^ plastic tissue culture flasks and cultured in cell culture medium (Minimum Essential Media (Sigma-Aldrich)) supplemented by 1% nonessential amino acids (NEAA), 1 mM sodium pyruvate, 10% FCS, streptomycin (100 *μ*g/mL), and penicillin (100 IU/mL). Cell suspensions were prepared and diluted target cell density (10 000 cells/well). Cells were added by pipette (80 *μ*L) into 96-well microtiter plates. Inoculates were allowed a preincubation period of 24 h at 37°C and 5% CO_2_ for stabilization. Fourfold dilutions, in 20 *μ*L aliquots, of the intended test concentration were added to the microtiter plate wells at time zero. All test compound concentrations were examined in duplicate. Incubation of the cells with the test compounds lasted for 72 h at 37°C and 5% CO_2_. At the end of the incubation period, the cells were assayed using MTT. Aliquots (10 *μ*L) of the MTT stock solution were pipetted into each well and incubated for further 2 h. After this incubation period the formazan produced was dissolved by the addition of 100 *μ*L/well of 10% aq SDS (pH 5.5), followed by a further incubation at 37°C overnight. The optical density (OD) was measured at 560 nm by EnSpire multimode plate reader (PerkinElmer). Cell survival (IC50) was calculated using the following equation: IC = (ODdrug-exposed well/mean ODcontrol wells) × 100%. The IC50 value, the drug concentration lethal to 50% of cells, was calculated from appropriate dose-response curves.

### 2.5. Luciferase Assays

BJ and HaCaT reporter cells were seeded in white 96-well plates (PerkinElmer) at concentration of 2 × 10^5^ in 100 *μ*L of Minimum Essential Media (Sigma-Aldrich) supplemented by 1% nonessential amino acids (NEAA), 1 mM sodium pyruvate, 10% FCS, streptomycin (100 *μ*g/mL), and penicillin (100 IU/mL) for 24 h at 37°C and 5% CO_2_. The media was replaced by Opti-MEM media (Gibco) containing 0.5% FBS and 1% NEAA and cells were treated by 40 *μ*g/mL of ginger extract or 30 *μ*M quercetin (Sigma-Aldrich) while the control wells contained media with corresponding concentration of the solvent 0.05% dimethyl sulfoxide (DMSO) as described by Bak et al. [17]. Following 10 h treatment, the cells were lysed by 20 *μ*L of cell culture lysis buffer (E153A, Promega) and 100 *μ*L of luciferase assay substrate (E1483, Promega) was added. Luminescence was measured by EnSpire multimode plate reader (PerkinElmer). The values from luminescence assays were normalized by values acquired from MTT (3-(4,5-dimethylthiazol-2-yl)-2,5-diphenyltetrazolium bromide) assays with the cells that were prepared simultaneously with the same treatment conditions as described by Yamazaki et al. [18]. All the assays were performed three times with five technical replicates for each treatment.

### 2.6. Western Blot Analyses

For western blot analyses of GSTP1 protein, BJ and HaCaT cells were grown to confluence in 100 mm petri dish and media were replaced by treatment media solutions with 40 *μ*g/mL of ginger phenylpropanoids or 30 *μ*M of quercetin while control dish contained media with 0.05% DMSO for 10 h treatments. The protein lysates were obtained by lysis of cells with NP-40 lysis buffer (1% NP-40, 10 mM Tris, pH 7.5, 150 mM NaCl, 0.05 *μ*M dithiothreitol, 1x phosphatase, and protease inhibitor cocktails (Roche)) following 30 min incubation on ice and its clarification by centrifugation at 14 000 g at 4°C for 10 min. Western blot analyses were performed according to procedures described by Hrabakova et al. [19]. The proteins (50 *μ*g) were resolved on a 12% SDS-PAGE gel prior to membrane transfer. Blots were incubated with rabbit polyclonal anti-GSTP1 antibody (Immunotech) at a concentration of 1 : 100 overnight at 4°C and the GSTP1 bands of 23 kDa were visualised using secondary antibody (Alexa Fluor 488 goat anti-rabbit IgG). The membrane was stripped by incubation in stripping buffer (2% SDS, 6.25 mM Tris-Cl pH 6.8, 100 mM *β*-mercaptoethanol) for 30 min at 50°C and washed five times using wash buffer (0.1% Tween 20, 1x TBS). The stripped blots were reprobed by mouse monoclonal anti-*β*-actin antibody (Sigma) at concentration 1 : 10 000 and the actin bands of 42 kDa were visualised by secondary antibody anti-mouse Ig FITC conjugate. The intensities of detected GSTP1 protein bands were quantified using Image J system and normalized by the intensity of actin bands. The data from three independent protein samples were used for analyses.

### 2.7. Statistical Analyses

The significant differences in production of signals between luciferase of treated Nrf2 reporter cells controls and the significant difference in intensity of downstream target protein bands of treated parental cells were determined by independent *t*-test with an alpha level of 0.025, following Bonferroni's correction. All analyses were performed with STATISTICA software package.

## 3. Results

### 3.1. Phenylpropanoid Components of Ginger Extract

**Figure 1:**
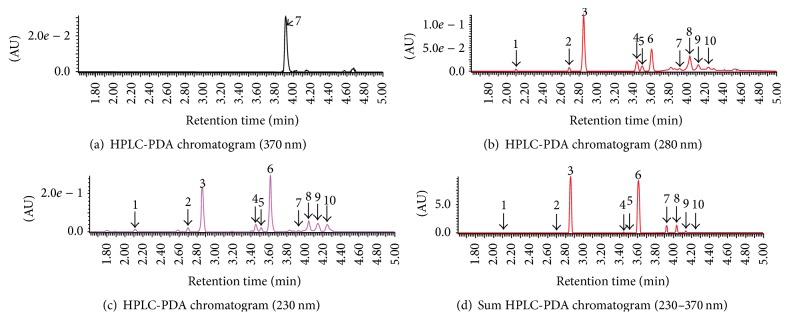
UHPLC-MS analyses of ginger methanol extract. (a), (b), and (c) are HPLC-PDA chromatograms of ginger methanol extract at 370, 280, and 230 nm, respectively. (d) is a Sum HPLC-PDA chromatogram in interval (230–370 nm) detected wavelength. All of the compounds were detected in negative ion mode ionization. Peaks with the identified phenylpropanoids are listed in Table 1. AU denotes absorbance units.

The ultrahigh performance liquid chromatography analyses of ginger extract identified compounds with absorbance at 230, 280, and 370 nm and molecular weights corresponding to those of ten different ginger phenylpropanoids: [6]-gingerol, [8]-gingerol, [10]-gingerol, methyl [6]-gingerol, [6]-gingerdiol, methyl 3- or 5-acetoxy-[6]-gingerdiol, methyl diacetoxy-[8]-gingerdiol, [12]-gingerdione, [8]-paradol, and [6]-shogaol (Figure 1, Table 1). All of the identified phenylpropanoids correspond to those of commercial powder of ginger extract that were described by Jolad et al. [16]. The two phenylpropanoids, [6]-gingerol and [6]-shogaol, were major constituents of the ginger extract, as the area under the peaks of these two compounds was 33.2% and 31.2%, respectively, while the other phenylpropanoids were present in low amounts (Figure 1).

### 3.2. Cytotoxicity of Ginger Phenylpropanoids and Quercetin on BJ and HaCaT Cells

Both ginger phenylpropanoids and quercetin showed limited cytotoxicity to BJ/HaCaT cells as their IC50 values were above maximum tested concentration 50 *μ*g/mL and 50 *μ*M, respectively.

### 3.3. Effects of Ginger Phenylpropanoids and Quercetin on Nrf2-ARE Pathway

**Figure 2:**
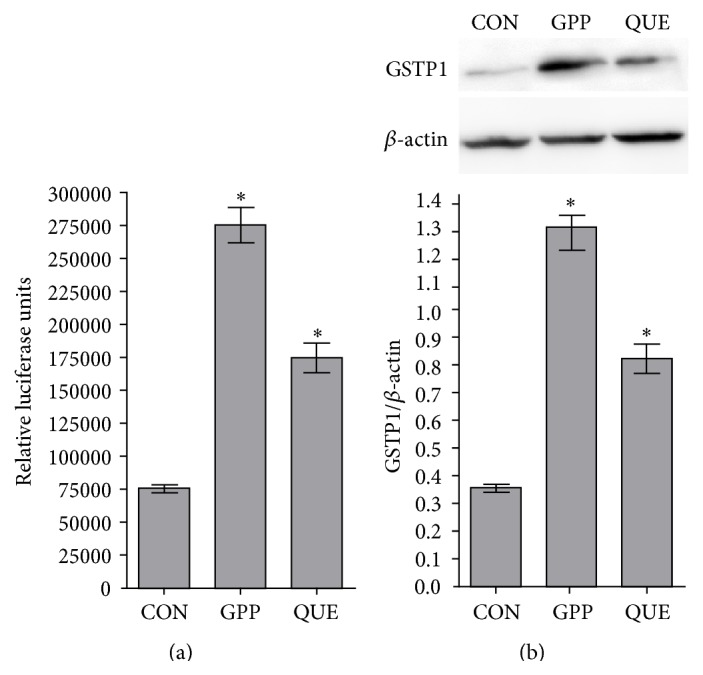
(a) Effects of ginger phenylpropanoids and quercetin on Nrf2 antioxidant pathway of luciferase ARE reporter BJ fibroblasts. (b) Western blot analysis of GSTP1 protein expression of treated BJ fibroblasts. CON, GPP, and QUE denote vehicle controls with 0.05% DMSO, ginger phenylpropanoids, and quercetin, respectively. In the graphs, *∗* means a statistically significant value from that of controls (*p* < 0.025). Western blots were done as three biological replicates.

**Figure 3:**
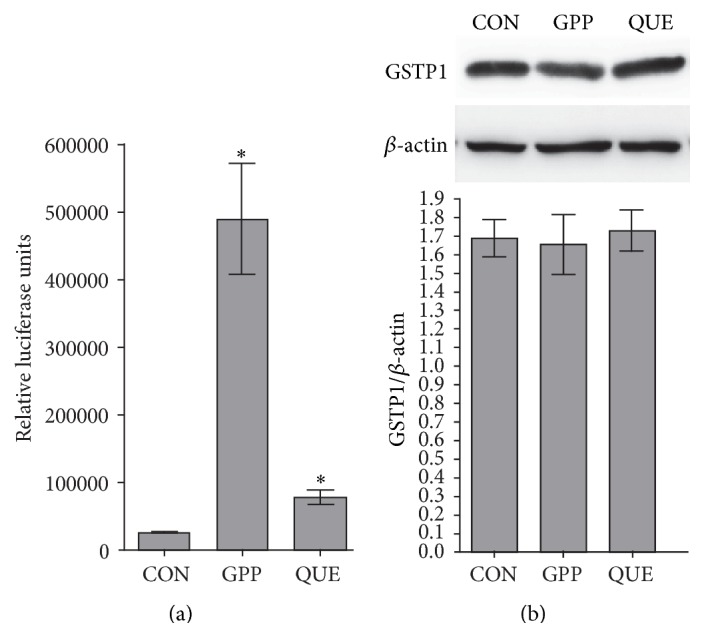
(a) Effects of ginger phenylpropanoids and quercetin on antioxidant pathway of luciferase ARE reporter HaCaT cells. (b) Western blot analyses of GSTP1 protein expression of treated HaCaT cells. CON, GPP, and QUE denote vehicle controls with 0.05% DMSO, ginger phenylpropanoids, and quercetin, respectively. In the graphs, *∗* means a statistically significant value from that of controls (*p* < 0.025). Western blots were done as three biological replicates.

Both ginger phenylpropanoids and quercetin had marked profound effects on luciferase activity of BJ and HaCaT ARE reporter cells. The average luciferase activity of BJ ARE reporter cells treated by ginger phenylpropanoids and quercetin was greater than that of their controls by 3.5 and 2.3 times, respectively (in both cases, *p* < 0.025, Figure 2(a)). The average luciferase activity of HaCaT ARE reporter cells treated by ginger phenylpropanoids and quercetin was greater than that of their controls by 4.9 and 19.9 times, respectively (in both cases, *p* < 0.025, Figure 3(a)). Western blot analyses showed that the average levels of GSTP1 protein in the BJ cells treated with ginger phenylpropanoids and quercetin were 6.5 and 2.6 times greater than that of their controls, respectively (in both cases, *p* < 0.025, Figure 2(b)). The average level of this protein did not differ significantly neither between the HaCaT cells treated by ginger phenylpropanoids and quercetin and that of their controls (*p* values for effects of ginger phenylpropanoids and quercetin were *p* = 0.79 and 0.87, Figure 3(b)). Comparing controls of HaCaT and BJ cells showed that its expression level was significantly higher in HaCaT cells (*p* < 0.025, Supplementary Figure  1, in Supplementary Material available online at http://dx.doi.org/10.1155/2016/2173275).

## 4. Discussion

Our analyses showed that both ginger phenylpropanoids and quercetin might have profound effects on Nrf2 signalling pathway in human skin cells. Both ginger phenylpropanoids and quercetin increased level of Nrf2 both in BJ and in HaCaT cells (Figures 2 and 3). These results are also consistent with previous studies that showed the effects of ginger phenylpropanoids and quercetin in the protection against oxidative stress in human skin cells [20–22]. Therefore, the studied ginger phenylpropanoids and quercetin have significant chemoprotective activity.

Specifically, in contrast to increased levels of both Nrf2 and its downstream target effector GSTP1 enzyme in BJ cells treated by ginger phenylpropanoids and quercetin (Figure 2(b)), the increased level of Nrf2 on treated HaCaT cells was not associated with an increased level of GSTP1 enzyme (Figure 3(b)). Such a discrepancy between the increased level of Nrf2 and its downstream effectors in HaCaT cells was also reported in previous studies of Zhang et al., [23] and it is considered that the unknown intrinsic factors might render* ARE* sequence of these genes unresponsive to Nrf2. Independent regulation of GSTP1 expression in different mouse embryonic tissues by Nrf2 inducers like ethoxyquin and butylated hydroxyanisole was also suggested by the* in vivo* work [24].

The Nrf2 independent regulation of GSTP1 expression in HaCaT cells might have selectively evolved with high proliferation capacity during immortalization. As immortalization is also a first step of carcinogenesis and a variety of human cancer cells including breast, colon, kidney, lung, and ovarian cancer cells share not only genomic instability, loss of senescence genes, mutation in p53 genes, and high proliferation rate but also the constitutively high expression of GSTP1 [25–27], the role of GSTP1 in HaCaT cells might be distinct from that of the normal cells. This is also supported by the fact that, in addition to its enzyme activity in the conjugation of reduced glutathione to a wide variety of electrophilic substrates, GSTP1 was also found to inhibit the two important nonsubstrate ligands by direct protein-protein interactions, c-Jun N-terminal kinase (JNK) and TNF receptor-associated factor 2 (TRAF2), a member of the TNF receptor-associated factor protein of JNK and p38-MAPK signalling complexes [28–30]. As these two MAP kinases are associated with signalling pathways of stress response and apoptosis [30–32], their inhibition by the high level of GSTP1 might be beneficial in inhibition of apoptosis and maintenance of high proliferation rate in HaCaT cells.

## 5. Conclusions

Both ginger phenylpropanoids and quercetin have property of activating the Nrf2 and expression of downstream target enzyme GSTP1 in BJ cells. On the other hand, while both ginger phenylpropanoids and quercetin can activate Nrf2 in HaCaT cells, their effects on expression of the GSTP1 were not mediated. This finding is in accordance with previous studies that showed that the ARE sequences in the promoter of GSTP1 gene are unresponsive to Nrf2 in HaCaT cells.

## Supplementary Material

The supplementary figure shows data on GSTP1 expression in controls of BJ fibroblasts and HaCaT cells.

## Figures and Tables

**Table 1:** Phenylpropanoids identified by UHPLC-MS.

Peak	Compound	Retention time (min)	MW	AUC (%)
1	[8]-Paradol	2.10	306	2.3
2	[6]-Gingerdiol	2.69	296	3.8
3	[6]-Gingerol	2.86	294	33.2
4	[8]-Gingerol	3.45	322	4.4
5	Methyl [6]-gingerol	3.51	308	3.0
6	[6]-Shogaol	3.61	276	31.2
7	Methyl 3- or 5-acetoxy-[6]-gingerdiol	3.92	352	7.3
8	[10]-Gingerol	4.03	350	7.8
9	Methyl diacetoxy-[8]-gingerdiol	4.13	422	4.7
10	[12]-Gingerdione	4.24	376	2.5

Note: AUC was calculated from Sum HPCL-PDA chromatogram (230–370 nm).
