# Supplementary material for: Effects of Ginger Phenylpropanoids and Quercetin on Nrf2-ARE Pathway in Human BJ Fibroblasts and HaCaT Keratinocytes
Source: Biomed Res Int. 2016 Jan 28;2016:2173275. doi: 10.1155/2016/2173275 (PMC4749771; doi:10.1155/2016/2173275)
Supplement: Supplementary file 1 — The supplementary figure shows data on GSTP1 expression in controls of BJ fibroblasts and HaCaT cells. [file 2173275.f1.pdf]

Supplementary material

Supplementary material includes a supplementary figure. The supplementary figure shows data on GSTP1 expression in controls of BJ fibroblasts and HaCaT cells.

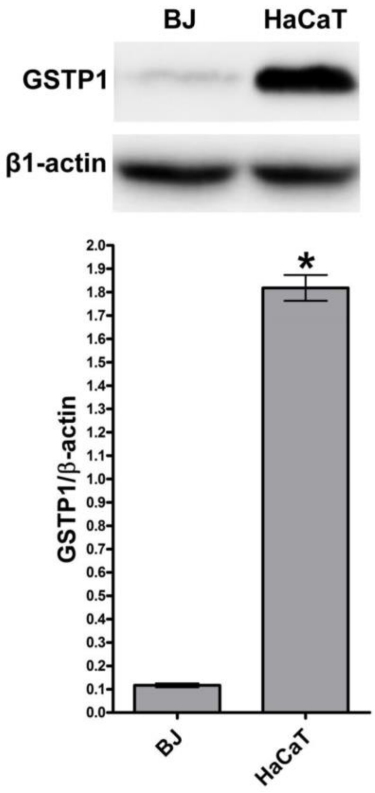

SUPLEMENTARY FIGURE 1: Western blot analysis of GSTP1 protein expression in controls of HaCaT and BJ cells. In the graph, * denotes statistically significant higher value from that of BJ controls ($p < 0.05$). Western blots were done as three biological replicates.
